# Occupational injuries and health risks among food delivery riders in China: research progress and prevention strategies

**DOI:** 10.3389/fpubh.2026.1932962

**Published:** 2026-08-13

**Authors:** Xueshun Xu, Xinran Ru, Ruxuan He, Zeya Shi

**Affiliations:** 1 School of Nursing, Hunan Normal University, Changsha, China; 2 Education Department, Kiang Wu Nursing College of Macau, Macau, Macao SAR, China; 3 Hunan Prevention and Treatment Institute for Occupational Diseases, Affiliated Prevention and Treatment Institute for Occupational Diseases of University of South China, Changsha, Hunan, China; 4 Jishou University School of Medicine, Jishou, Hunan, China

**Keywords:** algorithmic management, China, food delivery riders, gig economy, occupational health, occupational injury, road traffic injury

## Abstract

**Background:**

Platform-based on-demand food delivery has expanded rapidly in China, reshaping delivery work through strict deadlines, piece-rate pay, algorithmic management, long working hours, and intensive road exposure. These conditions may increase occupational injuries and broader health risks among food delivery riders.

**Objectives:**

This narrative review synthesizes evidence on occupational injuries and health risks among food delivery riders in China and identifies potential mechanisms and prevention strategies.

**Methods:**

We searched PubMed, Web of Science Core Collection, and CNKI for Chinese- and English-language literature published from January 1, 2015 to March 31, 2026. Search concepts included food delivery riders, platform work, occupational injuries, road traffic injuries, musculoskeletal disorders, fatigue, sleep, mental health, algorithmic management, and occupational health protection. Titles, abstracts, and full texts were screened for relevance, and 47 eligible sources were included.

**Results:**

Existing evidence indicates that riders face interrelated risks, including road traffic injuries, work-related musculoskeletal disorders, fatigue, sleep disturbance, occupational stress, anxiety and depressive symptoms, and chronic risks related to irregular eating and limited health management. Risky riding should be understood not only as an individual behavior, but also as a response to delivery deadlines, income pressure, algorithmic control, fatigue, road conditions, and inadequate protection. Key gaps include the predominance of cross-sectional studies, limited longitudinal and intervention evidence, insufficient subgroup analyses of full-time, crowdsourced, and part-time riders, and heavy reliance on self-reported exposure measures.

**Discussion:**

Occupational injuries and health risks among food delivery riders should be viewed as occupational and public health issues embedded in platform-based work organization. Prevention should move beyond individual safety education and post-accident compensation to include upstream governance of algorithms, delivery deadlines and working hours, fatigue and sleep management, traffic and ergonomic protection, accessible occupational health services, privacy-preserving monitoring, and multi-stakeholder support. Longitudinal and intervention studies are needed to clarify causal pathways and evaluate prevention strategies.

## 1 Introduction

The mobile Internet and platform economy have generated new forms of employment, with food delivery riders being a typical example. Occupational injury refers to physical injury, illness, or death caused by, or related to, work. Health risks refer more broadly to occupational exposures and potential consequences that may impair workers’ physical or psychological functioning. Against the backdrop of the rapid expansion of the platform economy, systematically assessing the occupational hazards and health risks faced by this emerging workforce has become an important concern in occupational and public health.

Previous studies have shown that workers in new employment models commonly face occupational injuries, work-related musculoskeletal disorders, mental health problems, and overwork (1–4). Food delivery riders have a dual identity as platform workers and road users. Their work is shaped by platform order allocation, route planning, customer ratings, and strict delivery deadlines. It also takes place mainly on urban roads, in commercial districts, in residential buildings, and outdoors. China’s food delivery market is large, highly platform-organized, and embedded in complex urban traffic environments. With the world’s largest food-delivery market, a large workforce of delivery riders, and a highly concentrated platform market, China represents one of the most important settings for studying occupational health issues in the platform economy (5).

In recent years, studies in China and elsewhere have increasingly examined road traffic injuries, long working hours, work-related stress, and mental health problems among food delivery riders (3, 4, 6–16). International evidence suggests that the health challenges associated with platform-based delivery work share common patterns across different settings. In a cross-sectional study of 425 food delivery riders in Tamil Nadu, India, high prevalences of occupational burnout, anxiety, and depressive symptoms were observed, and these outcomes were significantly associated with work-related factors such as monthly income, inadequate water intake, and skipping meals (17). Similarly, a survey of 709 motorcycle delivery riders in Chiang Mai, Thailand, found high levels of occupational hazard exposure, with work-related musculoskeletal disorders reported by 62.1% of participants (18). At the global level, gig workers commonly face the combined challenges of inadequate social protection and overlapping health risks (19, 20), while occupational safety and health risks associated with gig driving have also been documented in high-income countries (21).

Research from low- and middle-income countries further indicates that work organization can influence workers’ health by shaping lifestyle, physical activity, and sleep patterns. This relationship has been illustrated in studies of 420 workers at the YAZAKI plant in Kenitra, Morocco (22) and primary healthcare workers in South Africa (23). These international findings provide a broader context for understanding the occupational health challenges faced by Chinese food delivery riders and facilitate comparisons across occupational and socioeconomic settings. However, existing research remains largely focused on individual risk factors or isolated outcomes. The mechanisms through which platform rules affect riders’injuries and health through time pressure, fatigue, and high-risk riding behaviors remain insufficiently understood. Accordingly, this narrative review focuses on China and uses international studies as contextual and comparative evidence rather than as the basis for a global systematic review. Compared with previous reviews, its main contribution is to situate occupational injuries, physical and mental health risks, platform-based work organisation, and prevention strategies within a unified analytical framework, while highlighting the implications of China’s platform-delivery context for international occupational health governance.

To our knowledge, this is among the first China-focused narrative reviews to integrate occupational injuries, multidimensional physical and mental health risks, algorithmic management, platform-based work organisation, and prevention strategies among food delivery riders within a unified occupational health framework.

## 2 Methodology of the narrative review

This review searched the PubMed, Web of Science Core Collection, and CNKI databases for articles published between January 1, 2015, and March 31, 2026. The search terms focused on topics such as food delivery riders, platform work, occupational injuries, road traffic injuries, musculoskeletal disorders, fatigue, sleep, mental health, algorithmic management, and occupational health protection. The literature screening focused on occupational exposures, health outcomes, influencing factors, work organization, and prevention strategies for food delivery riders or similar platform-based delivery workers. Xue-Shun Xu and Xin-Ran Ru screened the literature based on titles, abstracts, and full-text content, ultimately including 47 eligible studies. Because this article is a narrative review rather than a systematic review, no meta-analysis was performed, and the review was not presented as PRISMA-compliant. Specific search and screening methods are presented in Table 1.

**Table 1:** Literature search and selection methods.

Item	Description
Review design	Narrative review of occupational injuries, health risks, associated factors, and preventive strategies among food delivery riders and comparable platform-based delivery workers.
Information sources	PubMed, Web of Science Core Collection, and CNKI.
Search period	Literature published from January 1, 2015 to March 31, 2026; last searched on March 31, 2026.
Search concepts	Food delivery riders, platform work, occupational and road traffic injuries, musculoskeletal disorders, fatigue, sleep, mental health, algorithmic management, occupational health, and prevention. Equivalent Chinese terms were used in CNKI.
Eligibility	Empirical studies, reviews, and—when directly relevant to the review topic—reports from international organizations, government policy or guidance documents, policy and legal analyses, expert commentaries, and empirical surveys published in trade periodicals were eligible; the latter categories served as supplementary evidence for background and policy discussion. News reports, unpublished materials, duplicate records, sources without extractable information, and publications unrelated to worker health were excluded.
Selection process	Titles, abstracts, and full texts were screened for relevance to occupational injuries, health risks, associated factors, and preventive strategies among food delivery riders and comparable platform-based delivery workers. Forty-seven eligible sources were included.

**Figure 1:**
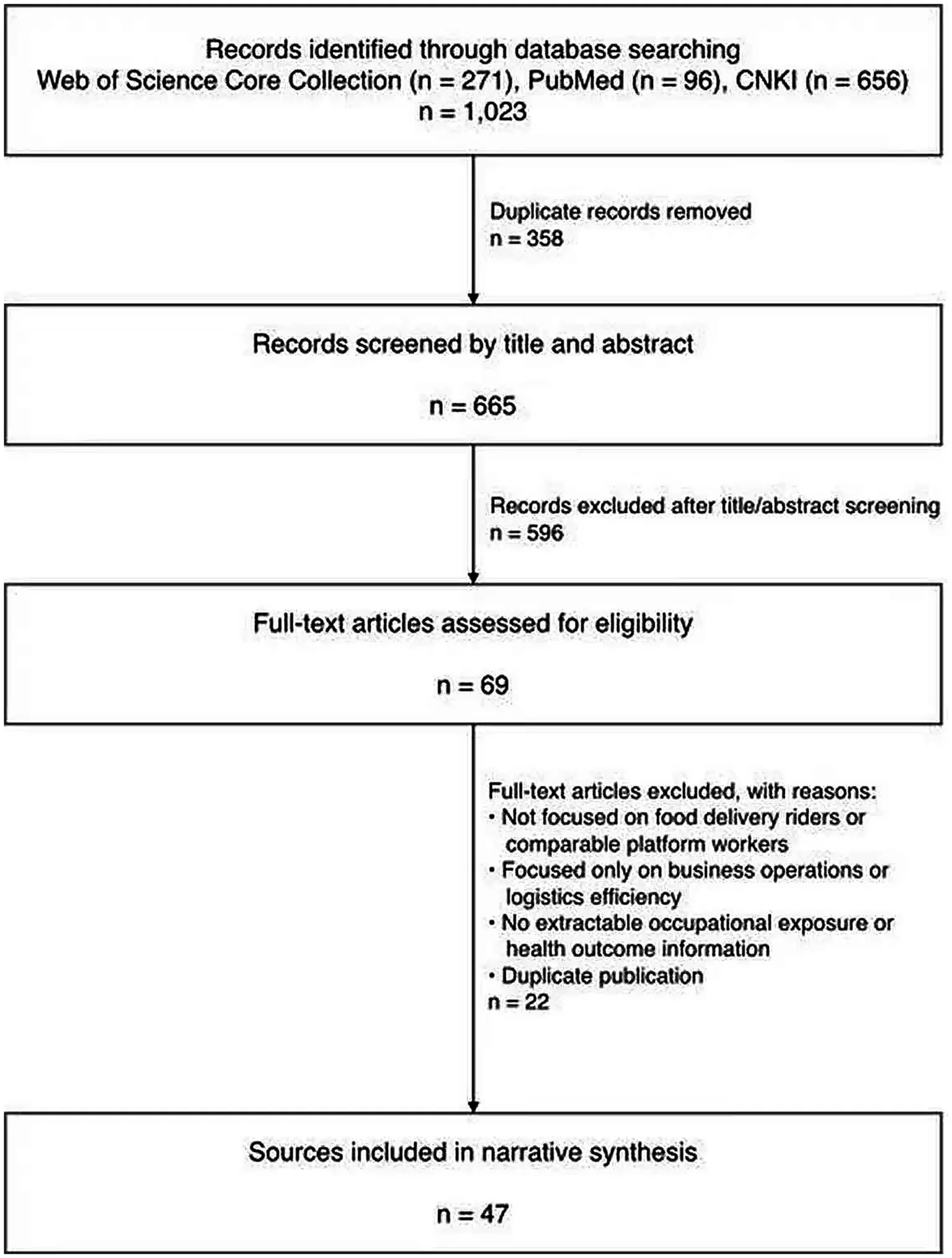
Flow diagram of the literature identification and selection process.

Literature selection proceeded in three stages. First, duplicate records and clearly irrelevant records were removed after database searching. Second, titles and abstracts were screened for relevance to occupational injuries, health risks, associated factors, work organisation, health behaviors, and prevention strategies among food delivery riders or comparable platform-based delivery workers. Third, full texts were assessed against the eligibility criteria. Full texts were excluded when they did not focus on delivery workers or comparable platform workers, addressed only commercial operation or logistics efficiency without worker-health information, lacked extractable information on occupational exposure or health outcomes, or represented duplicate publications. To further enhance methodological transparency, the complete selection process is summarized in Figure 1. Of the 1,023 records initially identified (PubMed, *n* = 96; Web of Science Core Collection, *n* = 271; CNKI, *n* = 656), 358 duplicate or clearly irrelevant records were removed, leaving 665 records for title and abstract screening. After the exclusion of 596 records at this stage, 69 full-text articles were assessed for eligibility, of which 22 were excluded for the reasons shown in Figure 1, resulting in 47 eligible studies included in the narrative synthesis. The selection logic is also summarized in Table 1.

## 3 Current status of occupational injuries and health risks among food delivery riders

### 3.1 Road traffic injuries

Road traffic injuries are the most prominent acute occupational injuries among food delivery riders. Riders mainly use electric bicycles or motorcycles and frequently move through urban roads, commercial districts, residential areas, and intersections. Their work involves long exposure times, complex traffic conditions, and a fast pace. Studies show that the most commonly injured body parts among on-demand delivery riders are the limbs, especially the lower limbs, followed by the head and face. Common injury types include abrasions, contusions, and fractures, and severe cases may lead to disability or death (7, 12). Regional surveys in China and international reviews further indicate that injury risk is associated with risky riding behavior, vehicle type, protective equipment, riding exposure, and working conditions (13–15).

Traffic injury risk is concentrated during peak order periods, night-time deliveries, adverse weather, congested road sections, and intersections. When strict platform deadlines coincide with traffic congestion, riders may speed, cut across traffic, or ride against the flow of traffic. Poor night-time visibility and slippery surfaces caused by rain or snow further increase the risk and severity of collisions and falls. Checking a mobile phone, accepting orders, or using navigation while riding can distract riders. Not wearing a helmet, riding in motor vehicle lanes, and running red lights are also associated with crash risk (7, 14, 15, 24). Prevention should therefore address high-risk behaviors while also identifying the work situations that trigger them.

Risky riding should not be explained simply as a lack of personal safety awareness. Work-related stress among on-demand delivery riders mainly stems from platform rules, penalties for late delivery, negative customer reviews, heavy workloads, and unstable employment relationships. These factors may increase road traffic injury risk through distraction, rushing, and risk-taking decisions (6). A study in Hangzhou showed that riders may still engage in high-risk riding even when they perceive a high crash risk, largely because of income pressure and strict time limits (9). Traffic injuries among food delivery riders therefore have clear occupational and organizational dimensions. They reflect the combined effects of platform management, income incentives, individual behavior, and the road environment.

### 3.2 Work-related musculoskeletal disorders and physical symptoms

Food delivery work involves multiple tasks, including riding, waiting for orders, carrying food, locating buildings, climbing stairs, and completing delivery. Prolonged fixed postures, frequent stops and starts, road vibration, load carrying, and repeated stair climbing can increase strain on the neck, shoulders, lower back, wrists, knees, and lower limbs. Relevant studies suggest that work-related musculoskeletal disorders among food delivery riders are associated with riding duration, years of service, and adverse ergonomic factors (24–26).

Food delivery riders also experience a range of physical symptoms beyond musculoskeletal disorders. A survey of 1,050 riders in Shenyang reported a physical symptom prevalence of 31.33%. The main symptoms were low back pain, wrist and knee pain, gastrointestinal symptoms, and muscle soreness. Older age, irregular eating habits, and poorer pre-employment health were identified as potential risk factors (11). These findings suggest that health risks among food delivery riders are not limited to acute injuries such as traffic crashes. They also include chronic physical discomfort and functional impairment that accumulate during long-term, high-intensity delivery work.

### 3.3 Long working hours, accumulated fatigue, and sleep problems

Long working hours and high-intensity labour are key factor linking platform management to a range of health problems. Piece-rate pay, requirements to remain online, and incentive–penalty mechanisms may encourage some riders to increase their earnings by working longer hours and taking fewer breaks. A survey conducted in Guangzhou found that the median weekly working time among food delivery riders was 63 h, and that 70.1% worked at least 55 h per week. The prevalence of occupational stress, depressive symptoms, insomnia symptoms, and cumulative fatigue was 30.1, 27.5, 34.7, and 40.8%, respectively. Working 55 h or more per week was significantly associated with occupational stress and cumulative fatigue (8). Other occupational epidemiological evidence also suggests that long working hours may increase the risk of work-related injuries and ill health (27, 28).

Peak orders, night-time deliveries, weather-related bonuses, and income targets can make work schedules irregular and reduce continuous rest and sleep. Sleep deprivation and accumulated fatigue may impair attention, judgment, and reaction time, thereby increasing distraction and risky riding. Persistent fatigue may also worsen musculoskeletal discomfort and negative emotions. These factors suggest a possible pathway from long working hours to sleep deprivation or fatigue, then to risky behavior, injury, or other health impairment. The direction and magnitude of these relationships still require longitudinal verification.

### 3.4 Work-related stress and mental health problems

Food delivery riders are exposed to multiple sources of stress, including time-based performance metrics, customer rating systems, restaurant delays, unpredictable traffic, changing weather, unstable income, and platform discipline. Previous reviews indicate that high work intensity, excessively long working hours, income volatility, algorithmic monitoring, and limited social support may increase the risk of anxiety, depression, and occupational burnout (3, 4, 10). A Shanghai study also found that symptoms of anxiety and depression were associated with work-related factors and individual characteristics (16).

Algorithmic management may affect mental health by reducing workers’ actual control over their work pace (29). Although riders can formally accept orders flexibly, order allocation, route planning, late-delivery judgments, and customer ratings continuously shape the work process. When riders cannot adjust their pace according to their physical condition, road conditions, or weather, they may experience helplessness, perceived unfairness, and anxiety. Because existing studies rely mainly on self-reported data, these mechanisms need further testing using platform rules, objective working-hour data, and longitudinal health information.

### 3.5 Health behaviors and chronic health risks

Irregular eating patterns, inadequate fluid intake, and limited access to healthcare resources are common work-related health concerns among food delivery workers. Because peak delivery periods often coincide with consumers’ mealtimes, riders may delay meals, eat hurriedly, or reduce their fluid intake and bathroom breaks to meet delivery deadlines. Prolonged exposure to these working conditions may be associated with gastrointestinal discomfort, changes in body weight and metabolic function, and the exacerbation of fatigue, sleep disturbances, and psychological stress (30, 31).

Research on health information-seeking behavior shows that most food delivery riders actively seek health information. They mainly rely on new media channels such as WeChat, Weibo, and short-video platforms, and their interests focus on disease knowledge, healthy eating, first aid, mental health, and sleep (32). This pattern suggests that riders have clear health needs, but conventional health education delivered at fixed places and fixed times does not fit their mobility or fast work rhythm. Future occupational health interventions should embed health communication, risk alerts, and service referral into platform apps, delivery stations, and riders’ daily work routes.

To synthesize the scattered evidence on occupational injuries, health risks, and their underlying mechanisms among food delivery riders, Table 2 summarizes the basic characteristics and key findings of the 47 included studies. This map systematically organizes key findings, risk pathways, and occupational health implications, providing a framework to support subsequent analyses of influencing factors and discussions on prevention and control strategies.

**Table 2:** Basic characteristics and main findings of included studies.

Author and year	Country	Study design	Sample characteristics	Main occupational exposures	Principal outcomes	Key findings
Liu et al. (2023) (1)	China	Cross-sectional survey	Workers in new forms of employment	Platform-based work organisation; occupational hazards	Occupational injuries and health status; protection needs	Workers in new forms of employment face the dual challenges of high risks and weak protection, highlighting the need for targeted protective measures.
Jing et al. (2023) (2)	China	Empirical study	Food delivery platform riders	Dependence on platform income; workload; platform rules	Work-related injury risk	Greater dependence on platform income and heavier workloads were associated with a higher risk of work-related injuries.
Margerison et al. (2025) (3)	International (scoping review)	Scoping review	Gig economy workers	Gig-work organisation; algorithmic management	Physical and mental health outcomes	Gig work was associated with multiple adverse health outcomes; evidence specific to food delivery platforms was limited and heterogeneous.
Matilla-Santander et al. (2025) (4)	International (scoping review)	Scoping review	Workers on location-based and online digital labour platforms	Platform-work exposures	Health status	Existing studies indicate poor mental health and adverse working conditions among platform workers; longitudinal evidence remains scarce.
Zhao et al. (2021) (5)	China	Industry analysis/ review	China’s food-delivery industry	Industry expansion; delivery work	Industry development; nutrition/public health context	China has the world’s largest food-delivery market, creating a large and representative rider workforce.
Song and Li (2025) (6)	China	Narrative review	Food delivery riders	Road-traffic exposure; work-related stress	Road traffic accidents; work-related stress	Work-related stress is closely associated with road traffic accidents among food delivery riders; organisational factors warrant attention.
Yang et al. (2024) (7)	China	Narrative review	Food delivery riders	Road environment; riding behavior	Traffic accidents and associated factors	Traffic accidents among riders are influenced by behavioral, vehicle, environmental, and work-related factors, calling for multilevel prevention.
He et al. (2023) (8)	China (Guangzhou)	Cross-sectional survey	Food delivery riders (median 63 h/week; 70.1% worked ≥55 h/week)	Long working hours; piece-rate pay	Occupational stress; depressive symptoms; insomnia; cumulative fatigue	The prevalences of occupational stress, depressive symptoms, insomnia, and cumulative fatigue were 30.1, 27.5, 34.7, and 40.8%, respectively; working ≥55 h/week was significantly associated with occupational stress and cumulative fatigue.
Shi et al. (2020) (9)	China (Hangzhou)	Cross-sectional survey	Food delivery riders	Income pressure; strict delivery deadlines	Accidental injuries; risk perception; protection needs	Despite perceiving a high risk of accidents, riders may engage in risky riding, mainly because of income pressure and delivery deadlines.
Cheng et al. (2026) (10)	China	Narrative review	Food delivery riders	Work-related stressors; platform management	Mental health status and associated factors	Riders’ mental health is influenced by workload, income instability, and platform management, underscoring the need for targeted interventions.
Bai et al. (2025) (11)	China (Shenyang)	Cross-sectional survey	1,050 food delivery riders	High-intensity delivery work; irregular eating	Somatic symptoms (low-back, wrist, and knee pain; gastrointestinal discomfort)	The prevalence of somatic symptoms was 31.33%; older age, irregular eating, and poorer health before entering the occupation were potential risk factors.
Useche et al. (2025) (12)	International (systematic review)	Systematic review	Last-mile delivery workers	Delivery-work organisation; time pressure	Occupational risks and safety outcomes	Last-mile delivery workers face elevated risks of occupational accidents, fatigue, and stress; organisational factors are key determinants.
Chen et al. (2019) (13)	China	Cross-sectional survey	Food delivery riders	Road-traffic exposure	Road traffic accidents and associated factors	Road traffic accidents are common among food delivery riders and are associated with riding behavior and work-related exposures.
McKinlay et al. (2022) (14)	International (scoping review)	Scoping review	Food delivery riders presenting with emergency-department injuries	Riding exposure; delivery work	Types of injuries and risk factors	The extremities, particularly the lower limbs, were the most common injury sites, followed by the head and face; abrasions, contusions, and fractures predominated.
Wang et al. (2021) (15)	China	Cross-sectional study	Electric-bicycle couriers and food delivery riders	Electric-bicycle riding; traffic environment	Road-safety behavior and accident involvement	Risky riding and inadequate use of protective equipment were associated with accident risk among delivery riders using electric bicycles.
Peng et al. (2022) (16)	China (Shanghai)	Cross-sectional survey	Food delivery workers	Work-related factors; individual characteristics	Anxiety and depressive symptoms	Anxiety and depressive symptoms were associated with work-related factors and individual characteristics.
Samal et al. (2025) (17)	India (Tamil Nadu)	Cross-sectional study	425 app-based food delivery riders	Work-related factors; inadequate fluid intake; skipping meals	Anxiety, depression, and occupational burnout	The prevalences of occupational burnout, anxiety, and depressive symptoms were high and were significantly associated with monthly income, inadequate fluid intake, and skipping meals.
Kwangsukstith et al. (2025) (18)	Thailand (Chiang Mai)	Cross-sectional survey	709 motorcycle food delivery riders	Occupational hazards; use of personal protective equipment	Work-related musculoskeletal disorders; health effects	Exposure to occupational hazards was high; the prevalence of work-related musculoskeletal disorders was 62.1%.
Bajwa et al. (2018) (19)	Global	Review	Gig economy workers	Non-standard employment; weak social protection	Health and well-being of gig workers	Gig workers globally face multiple overlapping health risks and inadequate social protection.
International Labour Organization (2021) (20)	Global	Global report	Digital labour platform workers (approximately 12,000 respondents)	Long working hours; income volatility; inadequate social protection coverage	Working conditions on digital labour platforms	Platform workers commonly face long working hours, substantial income volatility, and inadequate social protection coverage.
Christie and Ward (2019) (21)	High-income-country settings	Review/analysis	Drivers in the gig economy	Driving work; gig-work organisation	Occupational safety and health risks	Driving work in the gig economy involves substantial occupational safety and health risks, which are inadequately addressed by existing frameworks.
El Oirdi (2026) (22)	Morocco (Kenitra)	Cross-sectional study	420 workers at the YAZAKI automotive wire-harness plant	Work pace; shift systems	Lifestyle; physical activity; sleep	Work pace and shift schedules were closely associated with unhealthy lifestyles, insufficient physical activity, and sleep problems.
Mashita et al. (2025) (23)	South Africa	Cross-sectional study	174 primary healthcare workers	Work organisation; shift work	Physical-activity patterns and lifestyle habits	Work schedules were associated with insufficient physical activity and unhealthy lifestyle habits, illustrating the work organisation–lifestyle–health pathway.
Yang et al. (2021) (24)	China	Ergonomic survey	Food delivery workers in the catering industry	Ergonomic factors; riding duration; job tenure	Work-related musculoskeletal disorders (WMSDs)	WMSDs among delivery workers were associated with riding duration, job tenure, and adverse ergonomic factors.
He (2022) (25)	China	Review	Workers with work-related musculoskeletal disorders	Ergonomic exposures	WMSD prevention and control	There is an urgent need to strengthen ergonomic interventions and WMSD prevention strategies for high-risk occupational groups.
Alrashidi et al. (2026) (26)	International (systematic review and meta-analysis)	Systematic review and meta-analysis	Food delivery workers	Delivery-work exposures	Work-related musculoskeletal disorders	The prevalence of WMSDs among food delivery workers was high, supporting the need for ergonomic and organisational interventions.
Dembe et al. (2005) (27)	United States	Epidemiological analysis	The US working population	Overtime; long working hours	Occupational injuries and illnesses	Long working hours and overtime were associated with increased risks of occupational injuries and illnesses.
WHO and ILO (2021) (28)	Global	Joint global estimates report	The global working population	Long working hours and other occupational exposures	Burden of work-related diseases and injuries	Long working hours and other occupational exposures contribute substantially to the global burden of work-related diseases and injuries.
Sun (2023) (29)	China	Quantitative analysis	Online labour-platform workers	Algorithmic management	Workers’ mental health	Algorithmic management may harm workers’ mental health by weakening their actual control over the pace of work.
Wang S et al. (2025) (30)	China	Cross-sectional study	Workers in new forms of employment	Irregular eating; inadequate fluid intake; work patterns	Cardiovascular and metabolic risk factors	Cardiovascular and metabolic risk factors clustered among workers in new forms of employment and were associated with work patterns and lifestyle.
Feng et al. (2026) (31)	China	Quasi-experimental study with experience sampling	Ride-hailing drivers	Skipping meals; sleep deprivation	Driving-safety hazards	Skipping meals and sleep deprivation impaired attention and driving performance, illustrating a lifestyle-mediated occupational risk pathway.
Kang et al. (2024) (32)	China (Shanghai)	Cross-sectional survey	Food delivery riders	Health-information-seeking behavior	Sources and topics of health information	Most riders actively sought health information through new media, including WeChat, Weibo, and short-video platforms, focusing on disease knowledge, healthy eating, first aid, mental health, and sleep.
Griesbach et al. (2019) (33)	United States	Qualitative/ethnographic study	Platform-based food delivery workers	Algorithmic control	Work processes and control mechanisms	Platforms continuously regulate work processes through order allocation, route planning, time limits, ratings, and rewards, with limited transparency of the rules.
Wang (2023) (34)	China	Legal analysis	Platform-work governance	Platform-based employment relationships	Tripartite labour governance	A “three-part” governance model was proposed to clarify labour-protection responsibilities in platform work.
Chen et al. (2025) (35)	China	Empirical study	Gig workers	Algorithmic control	Self-depletion; safety performance	Algorithmic control had a dual effect on gig workers’ self-depletion and safety performance.
Papakostopoulos and Nathanael (2021) (36)	Greece (Athens)	Cross-sectional study	107 food delivery riders	Work-related factors; time pressure	Risky riding behavior	Work-related factors showed complex associations with risky riding behavior among food delivery riders.
Zheng et al. (2019) (37)	China	Cross-sectional survey	Food delivery riders in China	Working conditions	Traffic-accident involvement; risky riding behavior	Working conditions significantly influenced accident involvement and risky riding behavior among food delivery riders.
Wang et al. (2021) (38)	China	Observational/modelling study	Electric-bicycle food delivery riders	Familiarity with traffic regulations	Electric-bicycle accidents; helmet use	Greater familiarity with traffic regulations was associated with lower accident risk and higher helmet-use rates, as confirmed by an ordered logit mediation model.
Nguyen et al. (2023) (39)	Vietnam	Cross-sectional study	Food delivery riders in Vietnam	Road-traffic exposure; riding behavior	Non-fatal traffic accidents	Non-fatal traffic accidents were common among food delivery riders in Vietnam and were associated with both behavioral and environmental factors.
Kim et al. (2026) (40)	South Korea	Qualitative interview study	9 motorcycle food delivery workers	Delivery-work conditions	Health status and health management	Motorcycle delivery workers reported multiple health problems, while health management remained inadequate under current working arrangements.
Howard (2017) (41)	United States	Review	Workers in non-standard employment arrangements	Non-standard employment	Workers’ health and safety	Non-standard employment arrangements may limit access to prevention, emergency assistance, and rehabilitation, thereby increasing health and safety risks.
Ran and Zhao (2023) (42)	China	Policy/research analysis	Gig workers in China	Flexible employment; insurance gaps	Occupational injury protection	Gig workers in China face inadequate occupational injury protection behind nominal employment flexibility.
Ministry of Human Resources and Social Security (2023) (43)	China	Policy guideline	Workers in new forms of employment	Rights to rest and remuneration	Labour-rights protection guideline	The national guideline provides an institutional basis for protecting the rights to rest and remuneration of workers in new forms of employment.
Lu et al. (2022) (44)	China (Xi’an)	Case study	The food-service and delivery sector	Traffic violations	Reduction in traffic violations	A targeted intervention reduced traffic violations in the food-service and delivery sector.
Useche et al. (2024) (45)	Colombia	Cross-sectional study	248 food delivery riders	Stress-related factors; fatigue	Occupational accidents	Stress-related factors and fatigue significantly predicted occupational accidents among food delivery riders.
Shi et al. (2026) (46)	China	Intervention evaluation	Non-motorised road users	Traffic-safety education	Violations; heterogeneous effects	Traffic-safety education reduced traffic violations among non-motorised road users, although the effects were heterogeneous.
Boniardi et al. (2024) (47)	Italy (Milan)	Cross-sectional study	240 digital food-delivery platform riders	Platform-based delivery-work exposures	Occupational safety and health status	Platform delivery riders in Milan faced multiple occupational safety and health risks, supporting the need for targeted preventive measures.

## 4 Key factors associated with occupational injuries and health risks among food delivery workers

### 4.1 Platform-based work organization and time pressure

Platform-based work organization forms the upstream context for occupational risks among food delivery riders. Piece-rate pay tightly links income to order volume, online time, delivery efficiency, and customer ratings. Riders therefore have to balance income, safety, and rest on a continuing basis. Delivery deadlines, late-delivery penalties, incentives, and negative customer reviews affect work intensity and may also shape risk-taking decisions and health behaviors.

Algorithmic management further reinforces these time constraints. Platforms continuously regulate work through order dispatch, route planning, delivery time limits, ratings, and incentive systems, yet rules and appeal mechanisms are not always transparent. When restaurants are late in preparing meals, traffic is congested, weather is poor, or building layouts are complex, riders often absorb the resulting time loss themselves. Related research suggests that algorithmic control and platform employment practices can affect worker autonomy and the implementation of occupational safety and health responsibilities (33–35). Working conditions are also associated with risky riding behaviors (36, 37). Analyses of occupational risk should therefore distinguish between individual-level behaviors and upstream determinants at the platform organization level.

### 4.2 Road traffic and outdoor work environments

The open and constantly changing road traffic environment is a major external factor in occupational injuries among food delivery riders. Riders frequently navigate mixed motorized and non-motorized traffic, congested areas, intersections, commercial districts, residential neighborhoods, and high-rise building clusters. They must repeatedly stop and start, yield to pedestrians and vehicles, and locate destinations. Insufficient bike lanes, slippery surfaces, slopes, narrow roads, poor night-time lighting, and road construction may all increase the risk of crashes, falls, and collisions.

Vehicle-related factors also contribute to occupational injury (38). The safety performance of electric bicycles, maintenance and warranty status, braking reliability, delivery-box installation, and helmet use are closely related to riding safety. To improve delivery efficiency, some riders illegally modify their vehicles or install accessories such as rain covers and oversized delivery boxes, which may compromise handling stability and braking performance. International studies also indicate that traffic environments, vehicle conditions, personal protective equipment, and work organization interact to influence crash rates and injury severity (14, 15, 37, 39). Road and vehicle risks should therefore be analyzed together with platform management factors.

### 4.3 Individual health behaviors and occupational health literacy

Individual characteristics and health behaviors influence riders’ ability to recognize, respond to, and recover from occupational risks. Age, riding experience, educational attainment, traffic safety awareness, pre-existing health conditions, and health literacy may all affect safety behavior and health outcomes. Insufficient riding experience, limited knowledge of traffic regulations, mobile phone use while riding, failure to wear helmets as required, and ignoring fatigue warning signs may increase the risk of road traffic injury (7, 15).

Unhealthy behaviors are also an important source of chronic occupational health risk. Because riders’ work time and location are unpredictable, they commonly report irregular eating, inadequate fluid intake, poor sleep quality, and low use of preventive health services (40). A Shanghai study on health information access found that food delivery riders were willing to seek health information proactively, mainly through WeChat, Weibo, and short-video platforms. The topics of interest included disease prevention and control, balanced diets, first aid skills, and mental health (32). These findings suggest that riders do not lack health needs. Rather, they need occupational health education that better matches their work environment, schedule, and information-access habits.

### 4.4 Gaps in occupational health support and protection

Insufficient occupational health support and protection can magnify the consequences of risk. Food delivery riders include dedicated riders, crowdsourced workers, and part-time workers, who differ in employment relationships, insurance coverage, occupational injury liability, and access to health services. Existing research has described workers in new employment forms as facing “high risks and weak protections” (1). International gig-economy literature also shows that non-standard employment and inadequate social protection can undermine access to prevention, emergency assistance, and rehabilitation support (19, 41, 42). For food delivery riders in China, the more critical issue is how these protection gaps affect risk reporting, accident management, rehabilitation, and the ability to continue working.

Occupational health services for food delivery riders remain insufficient, including traffic safety training, fatigue management, psychological support, and regular health checkups. Because riders often depend heavily on delivery income, have limited access to medical care, and are covered by weak social protection systems, they may delay seeking professional help after pain, sleep problems, anxiety symptoms, or accident risks emerge. Gig-economy research confirms that non-standard employment relationships, inadequate social protection, and insufficient occupational support increase workers’ health risks (19, 41). At the same time, policy regulations on rest and compensation rights for workers in new forms of employment provide an institutional basis for occupational health protection (43). The focus of prevention should therefore shift from post-incident compensation to routine monitoring, early intervention, and continuous support.

## 5 Prevention and management strategies for occupational health risks among food delivery workers

### 5.1 Optimizing platform algorithms and work organization

Occupational health protection for food delivery riders should follow the principle of controlling risks at their source. The focus should shift from requiring individual compliance alone to improving platform-based work organization. When setting delivery time limits and performance metrics, platforms should consider restaurant preparation time, traffic conditions, weather, building environments, delivery distance, and safety requirements. This would reduce the transfer of uncontrollable time losses to riders.

Feasible measures include regular rest reminders, daily order limits, and cumulative working-hour alerts to reduce the gradual accumulation of overwork. Delivery time limits should be extended during severe weather, and safety alerts should be sent for high-risk routes and peak hours to reduce the transfer of environmental pressure to individual workers (35, 44). At the same time, platform evaluation systems should not focus only on speed and order volume. They should also include indicators related to safe behavior, adequate rest, and health protection.

### 5.2 Strengthening traffic safety protection and context-specific occupational health services

Road traffic injuries remain the most common occupational injury among food delivery riders, making traffic safety management a key prevention priority (45). Dangerous behaviors such as speeding, running red lights, riding against traffic, using mobile phones while riding, and not wearing helmets should be addressed through onboarding training, regular refresher courses, and behavioral interventions (46). Platforms and regulators can improve riders’ safety capacity through cycling safety courses, accident case education, helmet-use reminders, regular vehicle inspection, reflective equipment for night-time riding, warnings about hazardous road sections, and alerts for adverse weather.

Occupational health services should be integrated into riders’ daily work routines. Platform apps, delivery stations, rider rest stops, community health service providers, and occupational health institutions can be used to provide traffic injury prevention, fatigue and sleep management, psychological support, musculoskeletal protection, extreme-weather protection, and first-aid training. Given the high mobility of riders and the fragmented nature of their work schedules, short videos and platform messages can help disseminate risk information (32). However, health education should not substitute for improvements in work organization and social protection.

### 5.3 Establishing occupational health monitoring and multi-stakeholder support

Occupational health protection should shift from post-incident compensation to proactive risk identification. With privacy and data security safeguards in place, tiered alerts could combine continuous online time, night-time and adverse-weather deliveries, accident records, and self-reported health information (46). Delivery hubs and primary healthcare facilities could conduct simple screening for blood pressure, body weight, sleep, psychological stress, and musculoskeletal symptoms, and then refer high-risk riders for further support. The use of monitoring data, authorization procedures, and opt-out mechanisms should remain transparent to prevent health monitoring from becoming another form of performance control.

Sustained prevention requires collaboration among government agencies, platforms, delivery sites, healthcare institutions, labor unions, and social organizations (47). Government departments should refine occupational injury protection and occupational health service policies for new forms of employment. Platforms should assume responsibility for safety education, labor protection, accident assistance, and improvements in work organization. Communities and social organizations can provide rest areas, drinking water, charging stations, psychological support, and rights counseling. For crowdsourced and part-time riders, more flexible and portable forms of protection should be explored, with attention to service accessibility across delivery models and urban contexts.

Emergency care after injury, medical referral, rehabilitation follow-up, and return-to-work guidance should also be included in continuous management. Road accident handling involves multiple parties, including delivery platforms, traffic police, healthcare providers, and insurers. Unclear procedures may cause minor injuries to be overlooked, delay insurance claims, or lead riders to return to work too early. An integrated platform-side pathway is therefore needed, covering accident reporting, station assistance, medical assessment, insurance coordination, and rehabilitation guidance. Such a pathway would create a closed loop from pre-injury warning to emergency response and post-injury recovery (42).

## 6 Discussion

### 6.1 Main findings and mechanisms

This narrative review synthesises current evidence on occupational injuries and health risks among food delivery riders in China, drawing on international studies for comparative and contextual insights. The findings suggest that the health risks faced by this population should not be viewed as isolated phenomena or attributed solely to individual unsafe behaviors. Rather, they are likely shaped by the interplay of platform-based work organisation, outdoor working environments, prolonged high-intensity work, individual health behaviors, and inadequate occupational health and safety protections.

One potential mechanism highlighted by this review is the interaction between platform-imposed time pressure and complex road environments. This interpretation should be treated cautiously because the available evidence is based predominantly on cross-sectional studies. Such designs make it difficult to establish the temporal sequence between exposures and outcomes. Moreover, the heavy reliance on riders’ self-reported exposures and health status raises the possibility of common-method bias and reverse causation. Accordingly, the pathway proposed in this review—“platform rules–time pressure–fatigue and sleep deprivation–risky riding behaviors–injuries and adverse health outcomes”—should be understood as a plausible inference consistent with the existing evidence, rather than as an established causal relationship. Its direction and magnitude require verification through longitudinal and intervention studies. Delivery deadlines, piece-rate payment systems, customer-rating systems, late-delivery penalties, and algorithmic dispatching may encourage riders to work longer hours, shorten rest periods, and prioritise speed over safety. These pressures may contribute to cumulative fatigue, sleep deprivation, distracted riding, and other risky riding behaviors, thereby increasing the risk of road traffic injuries and other adverse health outcomes. Risky riding should therefore not be interpreted solely as an individual behavioral choice; it may also represent an occupational coping response shaped by organisational and economic pressures.

The health risks of food delivery riders are also multidimensional and interrelated. Road traffic injuries represent the most visible acute occupational harm. Musculoskeletal disorders, physical symptoms, fatigue, sleep problems, psychological distress, and unhealthy behaviors may accumulate over time. Long working hours and irregular schedules may exacerbate fatigue and sleep disturbances, which may subsequently impair attention and reaction time while riding.

Repetitive cycling in constrained postures, walking while carrying loads, exposure to vibration, and stair climbing may increase musculoskeletal strain. Meanwhile, stress arising from algorithm-driven management, income instability, and inadequate social support may further compromise mental health.

**Figure 2:**
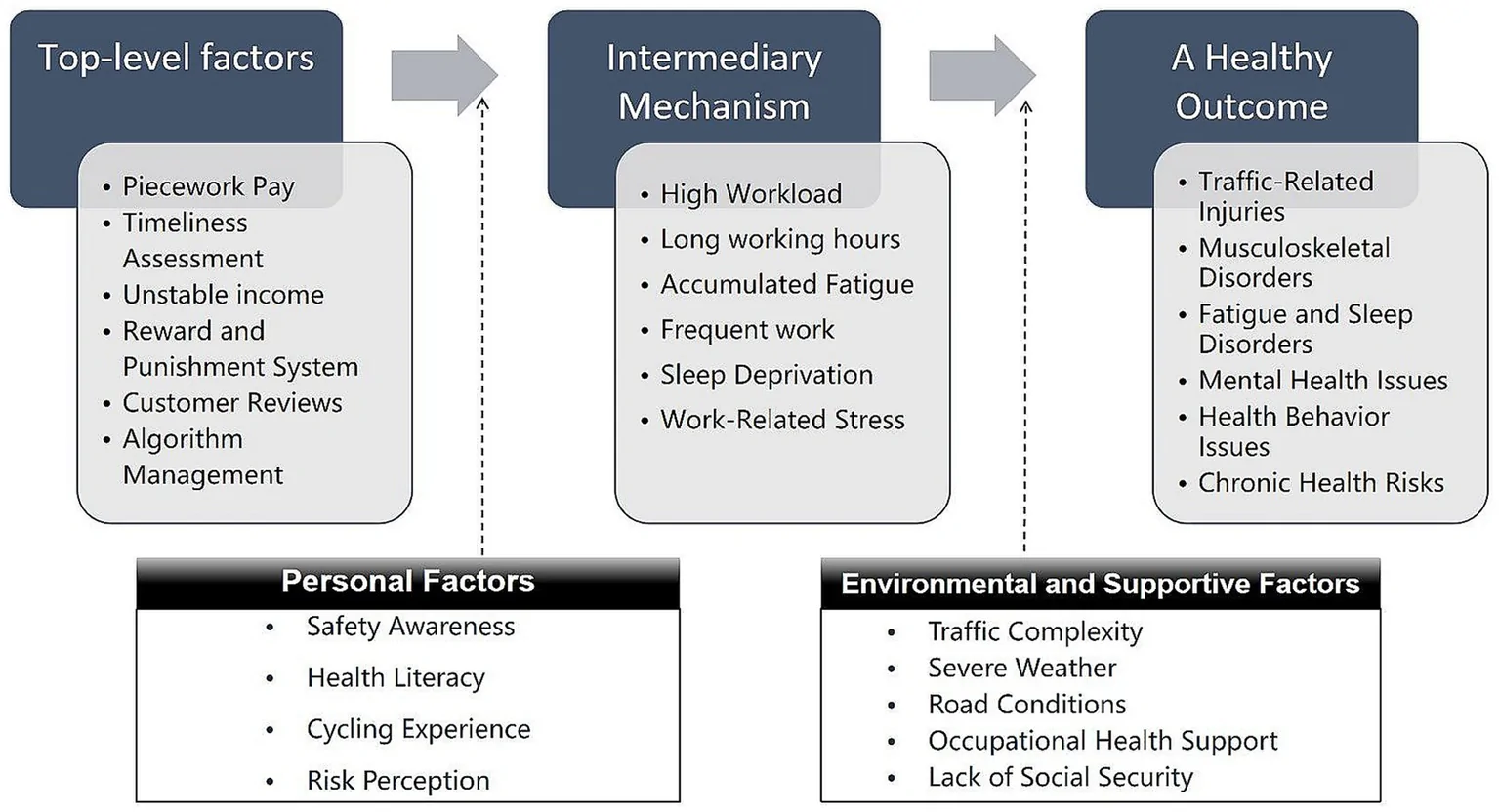
An integrated risk mechanism for platform management, work exposure, and health outcomes among food delivery riders in China.

Together, these mechanisms suggest that occupational injuries and health risks among food delivery riders should be assessed as an interconnected chain of risks rather than as isolated issues. Because cross-sectional studies account for a substantial proportion of the existing evidence, the mechanisms described above should be interpreted primarily as associations and potential pathways, rather than as fully established causal relationships. Figure 2 presents a conceptual framework illustrating this integrated chain of risks.

### 6.2 Public health and occupational health implications

The findings of this review have several implications for public health and occupational health practice. First, prevention should move from downstream compensation after accidents to upstream risk control. Although traffic safety education remains necessary, it is insufficient if platform rules continue to generate excessive time pressure. Platforms should consider restaurant preparation time, traffic congestion, weather conditions, building environments, delivery distance, and rider safety when setting delivery time limits and performance indicators. Rest reminders, cumulative working-hour alerts, severe-weather delivery adjustments, high-risk route warnings, and transparent appeal mechanisms may help reduce the transfer of organizational and environmental risks to individual riders.

Second, occupational health services for food delivery riders should be embedded into their actual work settings. Because riders are highly mobile and often work under fragmented schedules, traditional workplace-based health education may have limited reach. Platform applications, delivery stations, rider rest stops, community health service centers, occupational health institutions, labor unions, and social organizations can be used to provide more accessible services. These services may include traffic injury prevention, helmet and vehicle safety promotion, fatigue and sleep management, musculoskeletal protection, psychological support, first-aid training, and referral for high-risk riders.

Third, occupational health monitoring should be designed with caution. Data on online working time, night-time delivery, adverse-weather exposure, accident records, self-reported health status, and basic health screening could support early risk identification and stratified intervention. However, such monitoring should be based on clear rules for informed consent, privacy protection, data security, and opt-out options. Health-related monitoring should not become another form of performance control. For platform workers, occupational health protection must be linked not only to individual risk alerts, but also to fair work organization, social protection, and accessible health services.

Fourth, multi-stakeholder collaboration is essential. Government departments should improve occupational injury protection and occupational health policies for workers in new forms of employment. Platforms should assume greater responsibility for work organization, safety education, accident assistance, and health protection. Healthcare institutions can provide screening, referral, and rehabilitation support. Labor unions, communities, and social organizations can provide rest spaces, drinking water, charging facilities, rights counseling, and psychological support. For crowdsourced and part-time riders, more flexible and portable protection mechanisms are especially needed because their employment relationships and insurance coverage are often less stable.

### 6.3 International comparisons and implications for occupational health policies and the management of platform workers

Placing evidence from China in an international context shows that occupational health risks associated with platform-based delivery work share recurring patterns across settings, while also reflecting important structural differences. Studies from Greece (36), Vietnam (39), Colombia (45), Italy (47), South Korea (40), India (17), and Thailand (18) have reported associations between time pressure, income insecurity, and fatigue on the one hand, and traffic injuries, musculoskeletal symptoms, and psychological distress on the other. These findings suggest that such risks are not unique to China but may be linked to broader features of platform-based, on-demand delivery work organisation. The International Labour Organization’s global report on digital labour platforms likewise identifies excessive working hours, income volatility, and inadequate social protection as common challenges for platform workers (20). In high-income countries, gig workers engaged in driving occupations also face substantial occupational safety and health risks (21).

At the same time, structural differences between settings warrant careful consideration. In China, delivery riders primarily use electric scooters, urban population density is high, and platform markets are highly concentrated, with extensive algorithmic control over work organisation. Evidence from other low- and middle-income occupational settings further indicates that work organisation can influence health by shaping workers’ lifestyles, physical activity, and sleep patterns. For example, studies of 420 workers at the YAZAKI plant in Kenitra, Morocco (22) and of primary healthcare workers in South Africa (23) found that work rhythms and shift schedules were associated with unhealthy lifestyles, insufficient physical activity, and sleep problems. This broader evidence suggests that the irregular eating patterns, sleep deprivation, and longer-term health risks observed among Chinese delivery riders should be interpreted within a general “work organisation–lifestyle–health” framework rather than viewed solely as consequences of individual behavior.

These structural differences may help explain why algorithmic management appears particularly prominent in the Chinese context. The large scale and concentration of the food-delivery platform market, dense urban traffic, widespread use of electric scooters, and close linkage between income, order volume, delivery time, and customer ratings can make platform rules a direct driver of daily work intensity. In this setting, delays caused by restaurants, weather, congestion, or complex building layouts may be converted into individual time pressure borne by riders. This helps explain why fatigue, sleep loss, psychological stress, and injury risk may become tightly connected rather than separate outcomes. For other low- and middle-income countries undergoing rapid platform expansion, the Chinese case suggests that occupational health risks may emerge before social protection, road-safety infrastructure, and occupational health services have fully adapted to platform work. Therefore, prevention should address not only post-injury compensation but also the upstream design of platform rules, working-time control, and accessible occupational health services.

These findings have three implications for international occupational health policy and the management of platform workers. First, algorithmic management should be incorporated into occupational health risk assessments and relevant regulatory frameworks. Limited transparency regarding delivery deadlines, order allocation, and reward and penalty rules may constitute a modifiable occupational hazard (20, 33). Second, protections for platform workers should extend beyond workers’ compensation to include working-hours governance, fatigue and sleep management, mental health support, and accessible occupational health services. These protections should also account for precarious employment arrangements, including crowdsourced and part-time work (20, 21, 41). Third, comparative studies across countries with different income levels can help distinguish risks arising from the organisation of platform work itself from those shaped by local traffic conditions, labour markets, and social protection systems. Such evidence could support the development of context-sensitive yet internationally comparable occupational health standards.

### 6.4 Research gaps and future directions

Several research gaps remain. First, most available studies are cross-sectional or limited to single cities or regions, which restricts causal inference and generalizability. Many studies rely on self-reported exposure and health outcome data, while objective information on working hours, order intensity, riding distance, traffic environment, and weather exposure is still limited. Future studies should use longitudinal designs to clarify how platform rules, time pressure, fatigue, sleep problems, risky riding, and health outcomes influence one another over time.

Second, current studies often examine single outcomes, such as traffic injuries, musculoskeletal symptoms, psychological distress, or long working hours. Integrated research on co-occurring risks and shared mechanisms remains insufficient. Future research should examine how acute injuries and chronic health risks interact within the same occupational context. For example, fatigue may simultaneously increase crash risk, worsen musculoskeletal discomfort, and contribute to psychological distress. A more integrated framework would help identify high-risk subgroups and prioritize intervention targets.

Third, stronger attention should be paid to heterogeneity among food delivery riders. Dedicated riders, crowdsourced riders, and part-time riders may differ in working hours, platform dependence, income pressure, insurance coverage, accident reporting, and access to health services. Differences across cities, seasons, road environments, and platform management models should also be considered. Future studies should clearly define rider subgroups and explore whether risk mechanisms and intervention needs vary across these groups.

Fourth, intervention studies and effect evaluations remain limited. More evidence is needed on whether algorithmic optimization, rest policies, severe-weather protection, fatigue warning systems, traffic safety education, ergonomic interventions, psychological support, and embedded occupational health services can effectively reduce injuries and improve health outcomes. Future studies should also evaluate possible unintended consequences, such as whether health monitoring increases work surveillance or whether safety reminders are ineffective without changes in delivery time limits and income incentives.

Future research could integrate multiple data sources, including platform order data, online working time, riding trajectory, road and weather exposure, accident records, health questionnaires, physical examinations, and wearable device data. With appropriate privacy and ethical safeguards, these data could support interpretable risk prediction, early warning, and stratified intervention models. Mixed-methods studies would also be valuable, as qualitative interviews can help explain how riders perceive platform rules, health risks, and barriers to using occupational health services.

### 6.5 Limitations of this narrative review

Several limitations specific to this narrative review should be acknowledged. First, although the search covered PubMed, Web of Science Core Collection, and CNKI, the review was not designed as a systematic review and did not include a formal risk-of-bias assessment or quantitative meta-analysis. Second, the final evidence base included studies with heterogeneous designs, populations, exposure definitions, and outcome measures, which limits direct comparison across settings. Third, many included studies were cross-sectional and relied on self-reported exposures or health outcomes; therefore, causal pathways linking platform rules, time pressure, fatigue, and injury risk should be interpreted cautiously. Finally, because the review focuses on China and uses international studies mainly as comparative context, its conclusions may not be directly generalisable to all platform-delivery systems.

## 7 Conclusion

The occupational injuries and health risks faced by food delivery riders in China are associated with multiple factors, including the platform-based work organisation model, time pressure, road-traffic exposure, long working hours, constraints on health-protective behaviors, and inadequate occupational health and safety protections. The available evidence supports viewing these risks as occupational and public health concerns embedded in the platform-labour model, rather than merely as matters of individual safety awareness or riding behavior. However, because many studies are cross-sectional or limited to a single region, conclusions regarding causality and intervention effectiveness should be interpreted cautiously.

The following recommendations and inferences are based on the current evidence and require further empirical validation. Preventive efforts should extend beyond safety education and post-accident compensation to include upstream risk management and continuing support. Key priorities include algorithm and working-hours governance, traffic and ergonomic protection, and fatigue management. However, the effectiveness of these strategies is currently supported mainly by observational and indirect evidence, and evaluations of intervention outcomes remain limited. Similarly, the proposed pathway—“platform rules–time pressure–fatigue–risky riding–injury”—is theoretically plausible but has not yet been established as a causal chain. Differentiated protection strategies for full-time, crowdsourced, and part-time riders also require further refinement based on subgroup-specific research.

Future research should include longitudinal and intervention studies conducted across multiple regions and platforms. These studies should clarify the temporal sequence and strength of associations among platform rules, time pressure, fatigue, sleep, risky riding behaviors, and health outcomes, while also evaluating the effectiveness of preventive strategies such as algorithm optimization, rest policies, fatigue early-warning systems, traffic safety education, ergonomic protection, psychological support, and integrated occupational health services. Comparative research across countries and delivery models should also be strengthened to support the development of context-sensitive yet internationally comparable occupational health protection models for food delivery riders and other platform workers.

## Data Availability

Publicly available datasets were analyzed in this study. This data can be found at: the literature included in this review was sourced from the public databases PubMed and CNKI; the PMIDs and corresponding official website links for all included studies are fully listed in the references.
